# Clinical characteristics of acute Q fever patients in South Korea and time from symptom onset to serologic diagnosis

**DOI:** 10.1186/s12879-019-4479-0

**Published:** 2019-10-28

**Authors:** Jung Yeon Heo, Young Wha Choi, Eun Jin Kim, Seung Hun Lee, Seung Kwan Lim, Seon Do Hwang, Ju Young Lee, Hye Won Jeong

**Affiliations:** 1 Department of Infectious Diseases, Ajou University School of Medicine, Suwon, Republic of Korea; 2 Division of Bacterial Disease Research, Center for Infectious Disease Research, Korea National Institute of Health, Cheongju, Republic of Korea; 3 Yeosu National Quarantine Office, Korea Centers for Disease Control and Prevention, Yeosu, Republic of Korea; 4 Department of Internal Medicine, Gyeonggi Provincial Medical Center Ansung Hospital, Ansung, Republic of Korea; 5 Division of Bacterial Diseases, Center for Laboratory Control of Infectious Diseases, Korea Centers for Disease Control and Prevention, Cheongju, Republic of Korea; 6 Department of Internal Medicine, Sam Anyang Hospital, Anyang, Republic of Korea; 7 Division of Infectious Diseases, Department of Internal Medicine, Chungbuk National University Hospital, Cheongju, Republic of Korea; 8 Department of Internal Medicine, Chungbuk National University College of Medicine, Chungdae-Ro 1, Seowon-Gu, Cheongju, Chungbuk 28644 Republic of Korea

**Keywords:** Acute Q fever, IFA, Serologic diagnosis, Epidemiology

## Abstract

**Background:**

Acute Q fever usually presents as a nonspecific febrile illness, and its occurrence is rapidly increasing in South Korea. This study investigated the clinical characteristics of acute Q fever patients in South Korea and the time from symptom onset to serologic diagnosis. The clinical courses were examined according to antibiotic treatment.

**Methods:**

Data of patients diagnosed with acute Q fever at Chungbuk National University Hospital between January 2015 and February 2018 were retrospectively collected. Demographic and epidemiologic data were reviewed. The time from symptom onset to serologic diagnosis by an immunofluorescence assay (IFA) was analyzed. Clinical courses and the percentage of patients with a high phase I immunoglobulin G titer (≥ 1:1024) were compared between patients administered antibiotics with anti-*Coxiella burnetii* activity and patients not administered such antibiotics.

**Results:**

Forty-eight patients (median age: 51.5 years) were included. Most were male (95.8%) and had no history of animal contact (91.7%). The median time from illness onset to serologic diagnosis was 21 days. Thirty-nine patients received antibiotics with anti*-C. burnetii* activity. The length of hospital stay and fever duration did not significantly differ between patients who received antibiotics with anti-*C. burnetii* activity (7 and 15 days) and those who did not (5 and 8 days) (*P* = 0.110 and *P* = 0.137, respectively). The percentage of patients with a high phase I immunoglobulin G titer (≥ 1:1024) did not significantly differ between patients who received antibiotics with anti-*C. burnetii* activity and those who did not (*P* = 0.340).

**Conclusions:**

Most acute Q fever patients had a nonspecific febrile illness with mild elevation of transaminases and no history of animal contact or occupational risk. The time from symptom onset to a positive IFA test was longer than the fever duration in most acute Q fever patients. Consequently, it may be difficult for clinicians to serologically diagnose acute Q fever. However, inappropriate antibiotic treatment was not associated with prolongation of symptoms or progression to chronic Q fever.

## Background

Human acute Q fever, a zoonosis caused by the obligate intracellular bacterium *Coxiella burnetii*, presents as various clinical manifestations such as self-limited febrile illness, pneumonia, endocarditis, vascular infections, hepatitis, osteomyelitis, and meningoencephalitis [1]. Although humans can be infected through direct contact (e.g., ingestion or skin inoculation of contaminated animal products), the primary mode of transmission is via inhalation of dust contaminated with *C. burnetii* [2].

In 2006, Q fever was designated a notifiable infectious disease in South Korea. Thereafter, around ten cases of Q fever were reported annually until 2015. However, the occurrence of Q fever has increased in recent years, with 81 cases in 2016 and 96 cases in 2017. This corresponds to a larger than 6-fold increase compared with the 12 cases reported in 2008 [3]. Although Q fever has been detected in all regions of South Korea, with the exception of Jeju island, its incidence is highest in the Chungcheong region, which is located in the center of the country. Approximately 45% of all cases were reported in this region [3]. Until now, it is not clear that which factors are associated with the high incidence of human Q fever in Chungcheong area of South Korea. It was suggested that increasing number of raised goats in this region may have a major effect on the high incidence of Q fever [4]. Previous serologic and bacteriologic studies suggest that *C. burnetii* is extensively distributed among host animals in South Korea [5, 6]. Seroprevalence of Q fever in Korean cattle is 9.5–11.6% and seroprevalence in goats are 15–19% [6–9]. The seroprevalence of *C. burnetii* is 1.5% in healthy people and 10.2% in slaughterhouse workers [10, 11].

Q fever is mainly diagnosed by a serologic test and therefore paired serum samples are required for confirmatory diagnosis. This disease is thought to be underrecognized and underdiagnosed, particularly in non-endemic and non-epidemic areas such as South Korea, due to its nonspecific symptoms and challenging diagnosis. It is important to understand the clinical courses and timing of seroconversion in acute Q fever patients in order to appropriately manage and diagnose patients with a nonspecific febrile illness. Chronic Q fever develops in < 5% of patients with acute disease and is associated with serious complications such as endocarditis and vasculitis. Therefore, it is important not to misdiagnose acute Q fever patients who present with a nonspecific febrile illness when antibodies against *C. burnetii* are not detected [12, 13].

This study investigated the clinical characteristics of acute Q fever patients in South Korea and the time from symptom onset to serologic diagnosis. Furthermore, we compared the clinical characteristics of patients administered antibiotics with anti-*C. burnetii* activity and those not administered such antibiotics.

## Methods

### Study design and definitions

The medical records of patients diagnosed with acute Q fever at Chungbuk National University Hospital, which is a tertiary teaching hospital located in the Chungcheong region, from January 2015 to February 2018 were retrospectively reviewed. This hospital diagnosed more acute Q fever cases than any other institution in South Korea during the study period. The following data were collected: demographic data, epidemiologic data (living area, occupation, and history of animal contact), time to defervescence (the interval between the onset of fever and the first day when the patient’s peak fever had been lower than 37.3 °C for at least two consecutive days without antipyretics), length of hospital stay, clinical findings, antibiotic treatment, and serologic and laboratory test results. Cases with pneumonia were defined as those with consolidation on a chest X-ray or chest computed tomography scan. Cases with elevated transaminases were defined as those whose aspartate aminotransferase (AST) or alanine aminotransferase (ALT) levels were more than 3-fold higher than the upper normal limits in laboratory tests. Cases with positive autoantibodies were defined as those with an anti-nuclear antibody (ANA) or anti-neutrophil cytoplasmic antibody (ANCA) titer ≥1:80.

### Diagnosis of acute Q fever and analysis of clinical courses

Specimens of patients with suspected Q fever were sent to the Korea Centers for Disease Control and Prevention, where they were subjected to serologic testing for Q fever via an indirect immunofluorescence antibody (IFA) assay using a commercial kit (Focus Diagnostics, Cypress, CA, USA). Some specimens underwent PCR analysis as described in a previous study [14]. Acute Q fever was diagnosed based on the IFA or PCR results in patients with acute febrile illness. Cases with confirmed acute Q fever were defined as those with seroconversion to the phase II antigen, those in whom the phase II immunoglobulin G (IgG) titer differed by more than 4-fold between paired serum samples, or those with positive PCR or culture results with appropriate clinical findings. Cases with probable acute Q fever were defined as those with a phase II immunoglobulin M (IgM) titer of ≥1:16 or an IgG titer ≥1:256 in a single sample [2, 15, 16]. To investigate the serum antibody response to *C. burnetii* in detail, the time to serologic diagnosis was defined as the number of days from the onset of symptoms to the first positive result in the IFA test according to the aforementioned criteria.

Patients were categorized into two groups according to their treatment. Group 1 comprised patients who received antibiotics with activity against *C. burnetii* (tetracyclines, macrolides, quinolones, and rifampin) more than 3 days. Group 2 comprised patients who did not receive antibiotics with activity against *C. burnetii* more than 3 days. Time to defervescence, length of hospital stay, and the percentage of patients with a peak IgG titer ≥1:1024 were compared between the two groups.

### Statistical analysis

Demographic and clinical data were statistically analyzed using the Statistical Package for the Social Sciences (SPSS) for Windows, version 24 (IBM Corp., Armonk, NY, USA). Categorical variables were analyzed using Pearson’s χ2 and Fisher’s exact tests. The Mann-Whitney U test was used to compare continuous variables between the two groups. *P* < 0.05 was considered statistically significant.

### Ethics

This study was approved by the Institutional Review Board of Chungbuk National University Hospital (IRB No. 2012–03-024). The requirement for informed consent was waived because this was a retrospective study and there was no possibility of harming the enrolled subjects. All analyzed data were anonymized.

## Results

From January 2015 to February 2018, 203 febrile patients (120 males, 59%) were tested for Q fever via an IFA at Chungbuk National University Hospital. Among them, 51 patients (25.1%) were diagnosed with Q fever. Of these, 48 patients with acute Q fever (38 confirmed cases and 10 probable cases) were included in this study. PCR analysis of *C. burnetii* was performed in three cases, all of whom tested positive.

### Demographic and epidemiologic characteristics of acute Q fever patients

**Fig. 1:**
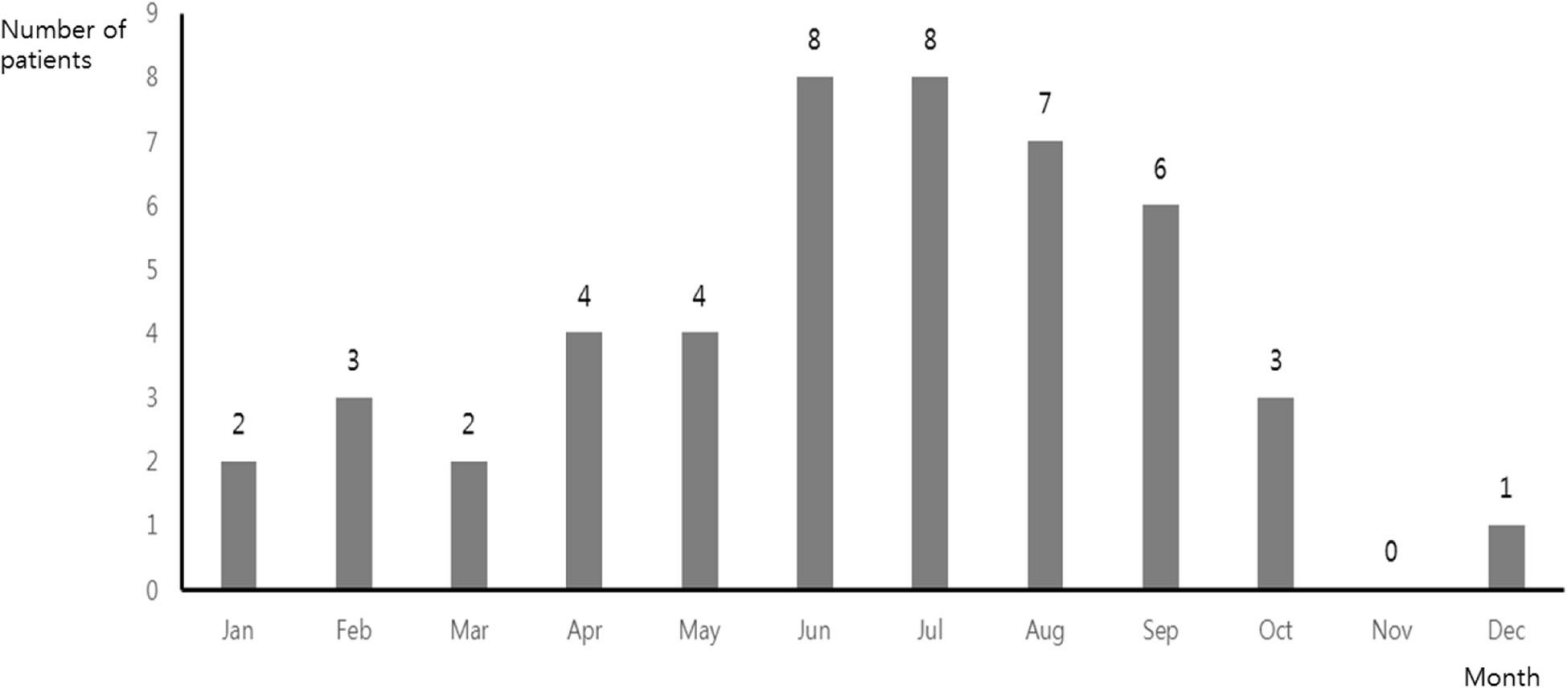
Number of patients who were diagnosed with acute Q fever in each month. Twenty-nine (60.4%) patients were diagnosed between June and September. In summer, animals are grazed in larger areas. People are more frequently exposed to the contaminated environments in farming season. It could be a reason of the slight increase of Q fever patients during summer

The median age of patients was 51.5 years [interquartile range (IQR): 46.3–58.8 years], and 46 (95.8%) patients were male (Table 1). In total, 27 (56.3%) patients had no underlying diseases, while 13 (27.0%) had hypertension and 11 (22.9%) had diabetes. Figure 1 shows the number of patients serologically diagnosed with acute Q fever in each month. Twenty-nine (60.4%) patients were diagnosed between June and September.

**Table 1:** Demographic and epidemiological characteristics of acute Q fever patients

	Acute Q fever patients
	*n* = 48
Male, n (%)	46 (95.8)
Age, years, median (IQR)	51.5 (46.3–58.8)
Underlying diseases	
No comorbidity, n (%)	27 (56.3)
Hypertension, n (%)	13 (27.1)
Diabetes mellitus, n (%)	11 (22.9)
Congestive heart failure, n (%)	2 (4.2)
Chronic liver disorder, n (%)	1 (2.1)
Cerebrovascular disorder, n (%)	1 (2.1)
Malignancy, n (%)	3 (6.3)
Animal contact, n (%)	4 (8.3)
Live in rural area, n (%)	24 (50.0)
Occupational risk, n (%)	7 (14.6)
Livestock raiser, n (%)	3 (4.2)
Veterinarian, n (%)	1 (2.1)
Farmer, n (%)	6 (8.3)

In relation to epidemiologic factors associated with Q fever, four (8.3%) patients had direct contact with animals due to their occupation (livestock raisers and veterinarians). These patients reported contact with goats (two cases), cattle (one case), and deer (one case). In total, 24 (50.0%) patients lived in rural areas and 6 (12.5%) patients were farmers; however, none of these patients reported any direct animal contact, except with companion dogs, or living near a barn. Thus, the majority of acute Q fever patients were previously healthy adults who lived in rural areas and lacked any known risk factors or underlying comorbidities.

The median time from illness onset to seeking of medical attention was 6.5 days (IQR: 4.0–14.0 days). The hospitalization rate was 89.6% and the median hospital stay was 6.5 days (IQR: 3.0–10.0 days). Most patients had a nonspecific acute febrile illness without localizing symptoms. Eleven (22.9%) patients had elevated transaminases and five (10.4%) patients had pneumonia. One patient had pericarditis. Table 2 summarizes the initial laboratory test results. Most patients exhibited mildly elevated C-reactive protein and transaminase levels. In total, 16 (66.4%) of 24 patients tested positive in autoantibody (ANA or ANCA) tests (Table 2).

**Table 2:** Clinical and laboratory findings of acute Q fever patients

Symptoms and laboratory findings	
Symptoms	
Fever, n (%)	48 (100)
Myalgia, n (%)	44 (91.7)
Headache, n (%)	30 (62.5)
Cough, n (%)	11 (22.9)
Joint pain, n (%)	7 (14.6)
Rash, n (%)	5 (10.4)
Pneumonia, n (%)	5 (10.4)
Elevated transaminases (> 3-fold higher than the upper normal limits), n (%)	11 (22.9)
Autoantibody tests performed, n (%)	24 (50.0)
Tested positive for autoantibodies, n (%)	16 (66.6%)
Initial laboratory test results	
White blood cell count (/μl), median (IQR)	5.98 (4.99–9.27)
Leukopenia (< 4000/μl), n (%)	2 (4.1)
Leukocytosis (> 10,000/μl), n (%)	10 (20.8)
Platelet count (× 1000/μl), median (IQR)	169.0 (133.0–247.5)
Thrombocytopenia (< 140,000/μl), n (%)	15 (31.2)
CRP (mg/dL), median (IQR)	7.7 (5.2–11.2)
AST (IU/L), median (IQR)	72.0 (50.0–98.7)
ALT (IU/L), median (IQR)	76.5 (53.2–97.5)
Elevated ALT (> 40 IU/L), n (%)	41 (85.4)
Elevated transaminases (> 3-fold higher than the upper normal limits), n (%)	11 (22.9)
Bilirubin (mg/dL), median (IQR)	0.65 (0.47–0.95)
Interval between illness onset and seeking of medical care, days, median (IQR)	6.5 (4.0–14.0)
Hospitalization, n (%)	43 (89.6)
Length of hospital stay, days, median (IQR)	6.5 (3.0–10.0)
Time to defervescence, days, median (IQR)	10.0 (7.0–22.8)
Time to serologic diagnosis, days, median (IQR)	21.0 (15.0–40.0)

### Serologic diagnosis of acute Q fever

The median time from illness onset to serologic diagnosis was 21 days (IQR: 15–40 days). Among the 48 patients, 40 (83.3%) underwent more than one IFA test and eight were diagnosed based on the results of a single IFA test (probable cases). Seventeen (35.4%) patients tested negative in the first IFA test. However, 15 (88.2%) of these patients tested positive in the second follow-up IFA test, while two patients (11.8%) tested negative. Of these two patients, one tested positive in the third follow-up IFA test, while the other tested negative. The latter patient tested negative in the fourth IFA test. The median time from illness onset to the first, second, third, and fourth IFA tests was 14, 45, 144, and 248 days, respectively.

A total of 115 IFA tests were performed in the 48 patients as diagnostic work-up or follow-up of antibody titers. To analyze the serologic test results in more detail, we categorized the results according to the week in which testing was performed after illness onset. Twenty-six IFA tests were performed within 2 weeks of illness onset, 12 (46.2%) of which yielded positive results. Therefore, only 12 of the 48 patients were diagnosed with acute Q fever within 2 weeks of illness onset. Twenty-nine IFA tests were performed during the third and fourth weeks after illness onset, 25 (86.2%) of which yielded positive results. Twelve IFA tests were performed during the fifth, sixth, and seventh weeks after illness onset, all (100%) of which yielded positive results. A patient who was diagnosed with acute Q fever based on detection of *C. burnetii* by PCR analysis tested negative in serial IFA tests up to 124 days after illness onset. With the exception of one case, all IFA tests performed during the eighth week after illness onset yielded positive results.

### Clinical courses of acute Q fever patients according to antibiotic treatment

Among the 48 patients, 39 received antibiotics with activity against *C. burnetii* more than 3 days (group 1) and the other nine did not (group 2). We compared the clinical courses of patients between these two groups. The median time to defervescence did not significantly differ (*P* = 0.137) between group 1 (15 days, IQR: 7–24 days) and group 2 (8 days, IQR: 6.5–10.5 days). The median hospital stay did not significantly differ (*P* = 0.110) between group 1 (7 days, IQR: 3–10.5 days) and group 2 (5 days, IQR: 3.5–6 days). The percentage of patients with a phase I IgG titer ≥1:1024 in serial IFA tests did not significantly differ between the two groups (6/39, 15.4% vs 3/9, 33.3%, *P* = 0.340).

## Discussion

We analyzed the clinical and epidemiological characteristics of patients diagnosed with acute Q fever between January 2015 and February 2018 in South Korea. The occurrence of Q fever increased rapidly during the study period [3]. The median age of patients was 51.5 years, and the majority of patients were previously healthy men who lived in rural areas and had no history of animal contact or occupational risk. Their clinical manifestations were nonspecific febrile illness. Due to these non-distinguishing clinical features and the lack of known risk factors such as animal contact, Q fever was underdiagnosed and underrecognized. The infection sources in this area are unclear. A further epidemiologic study including animals and environments might help to determine the origin of the infection.

In this study, 95.8% of the Q fever patients were male. In a survey on the 65 Korean Q fever patients reported in the national notifiable diseases surveillance system from 2006 to 2011, 57 patients were male (87.7%) [17]. Male predominance was also found in other study on Q fever of Australia [18] and in seroprevalence studies of the Netherlands and South Korea [19–21]. This gender imbalance is largely attributed to differential exposures to infected animals and contaminated environments thorough occupation. In addition to the different exposure risk between male and female, female sex hormone has some protective effect in Q fever [22–24]. This can potentiate gender disproportion of Q fever.

The time from illness onset to serologic diagnosis based on an IFA test (median: 21 days) was longer than the fever duration (median: 10 days). This is in contrast with patients with other rickettsial diseases, which usually show seroconversion in diagnostic tests within 7–10 days of symptom onset [25, 26]. The median time from illness onset to seeking of medical care was 6.5 days. Therefore, clinicians may fail to suspect and diagnose Q fever at an early stage in acute febrile patients. In this study, 35.4% of initial IFA tests yielded negative results, and these patients were diagnosed by follow-up tests at a late stage when they usually lacked clinical symptoms. To diagnose Q fever in non-endemic areas where this disease is underrecognized, such as South Korea, clinicians should suspect Q fever in patients with a nonspecific febrile illness who live in rural areas and should be aware of the delayed seroresponse.

After primary infection of *C. burnetii*, around 60% of patients are asymptomatic and the remainder display a fever and varying degrees of pneumonia or hepatitis [27, 28]. The major clinical manifestations of acute Q fever, such as hepatitis and pneumonia, differ between countries. Hepatitis is more frequently observed than pneumonia in France, southern Spain, and Taiwan [29–31], while pneumonia is the most prevalent manifestation in Nova Scotia in Canada, northern Spain, and the Netherlands [32–34]. This geographical variation might be due to differences in the route of infection, host factors, the infectious dose, and the strain of *C. burnetii* [27, 35–37]. In this study, 10.4% of patients had pneumonia and 22.9% of patients had elevated transaminases (more than 3-fold higher than the upper normal limits). Moreover, 85.4% of patients had an ALT concentration ≥ 40 U/L. Elevation of transaminases seems to be a more common clinical manifestation of acute Q fever than pneumonia in South Korea. However, a further study including more pneumonia patients is required to investigate the prevalence of *C. burnetii* in such patients because most patients included in the current study had a nonspecific febrile illness. Although hepatitis was the most prevalent feature of acute Q fever in this study, the AST and ALT concentrations were only modestly elevated (2–3-fold higher than the upper normal limits) in these patients. Autoantibody tests were performed as work-up in patients with a fever of unknown origin, 50% of whom tested positive. Immune reactions elicited by *C. burnetii* can produce various autoantibodies against cardiolipin, nuclear antigens, and smooth muscle antigens [38, 39]. In infective endocarditis, ANCA is associated with a longer duration of symptoms prior to diagnosis, and may result in multiple valve involvement and more frequent renal impairment [40]. Although it is unclear whether *C. burnetii* infection induces an autoimmune mechanism, circulating immune complexes might play a key role in the pathogenesis or severity of acute Q fever and lead to prolongation of fever, as observed in infective endocarditis.

Due to the considerable amount of time between illness onset and serologic diagnosis, diagnosis of Q fever and initiation of effective antibiotic treatment are often delayed. However, in this study, the time to defervescence and the hospital stay did not differ between patients who received antibiotics with anti-*C. burnetii* activity more than 3 days and those who did not. Other studies reported that doxycycline treatment significantly shortens the duration of fever in acute Q fever patients [41, 42]. It is likely that some acute Q fever patients have a self-remitting clinical course, while others have a protracted febrile illness that requires antibiotic treatment. Treatment of acute Q fever is not routinely recommended in asymptomatic cases or after resolution of symptoms [12]. A previous study reported that a phase I IgG antibody titer ≥1:800 at 3 and 6 months after illness onset is associated with chronic Q fever [43]. On the other hand, Wielders et al. demonstrated that early diagnosis and treatment of acute Q fever does not prohibit phase I IgG responses [44]. We analyzed whether inappropriate treatment of acute Q fever influences progression to chronic disease by assessing phase I IgG titers. The percentage of patients with a phase I IgG titer ≥1:1024 did not significantly differ between patients administered antibiotics with anti-*C. burnetii* activity and those not administered such antibiotics. Our results suggest that early initiation of appropriate antibiotic treatment does not affect the severity and duration of acute Q fever or progression to chronic Q fever.

Given the time delay and difficulties associated with serologic diagnosis and isolation of *C. burnetii*, PCR is an alternative option to diagnose acute Q fever within 2 weeks of illness onset [14]. In particular, real-time PCR analysis of *IS1111* is a useful diagnostic tool in acute Q fever patients that are seronegative and only display phase II IgM [45]. In the current study, PCR analysis was performed in three patients, all of whom tested positive. One patient with positive PCR in this study showed negative IFA tests on day 22, 56, 75 and 128 from illness onset. The PCR result of this case could be a false positive or we could not detect the serologic change of the patient due to the relatively long IFA test intervals. A further study is required to compare the diagnostic accuracies of the IFA and PCR analysis in acute Q fever patients in South Korea.

## Conclusions

The majority of patients diagnosed with acute Q fever were previously healthy males who lived in rural areas and presented with non-localizing febrile illness and mild elevation of transaminases. Serologic diagnosis of acute Q fever was usually achieved 3–4 weeks after illness onset. Late diagnosis and inappropriate antibiotic treatment were not associated with prolongation of acute Q fever or the development of chronic Q fever. These results provide baseline epidemiologic, clinical, and serologic data of acute Q fever patients in South Korea, a non-endemic area where this disease is underrecognized.

## Data Availability

The datasets used and/or analysed during the current study are available from the corresponding author on reasonable request.

## Abbreviations

ALT: Alanine aminotransferase
ANA: Anti-nuclear antibody
ANCA: Anti-neutrophil cytoplasmic antibody
AST: Aspartate aminotransferase
*C. burnetii*: *Coxiella burnetii*
IFA assay: Indirect immunofluorescence antibody assay
IgG: Immunoglobulin G
IgM: Immunoglobulin M
IQR: Interquartile range
PCR: Polymerase chain reaction
